# The Scaffolding Protein Dlg1 Is a Negative Regulator of Cell-Free Virus Infectivity but Not of Cell-to-Cell HIV-1 Transmission in T Cells

**DOI:** 10.1371/journal.pone.0030130

**Published:** 2012-01-17

**Authors:** Patrycja Nzounza, Maxime Chazal, Chloé Guedj, Alain Schmitt, Jean-Marc Massé, Clotilde Randriamampita, Claudine Pique, Bertha Cecilia Ramirez

**Affiliations:** 1 INSERM, U1016, Institut Cochin, Paris, France; 2 CNRS, UMR8104, Paris, France; 3 Université Paris Descartes, Sorbonne Paris Cité, Paris, France; University of Cape Town, South Africa

## Abstract

**Background:**

Cell-to-cell virus transmission of Human immunodeficiency virus type-1 (HIV-1) is predominantly mediated by cellular structures such as the virological synapse (VS). The VS formed between an HIV-1-infected T cell and a target T cell shares features with the immunological synapse (IS). We have previously identified the human homologue of the Drosophila Discs Large (Dlg1) protein as a new cellular partner for the HIV-1 Gag protein and a negative regulator of HIV-1 infectivity. Dlg1, a scaffolding protein plays a key role in clustering protein complexes in the plasma membrane at cellular contacts. It is implicated in IS formation and T cell signaling, but its role in HIV-1 cell-to-cell transmission was not studied before.

**Methodology/Principal Findings:**

Kinetics of HIV-1 infection in Dlg1-depleted Jurkat T cells show that Dlg1 modulates the replication of HIV-1. Single-cycle infectivity tests show that this modulation does not take place during early steps of the HIV-1 life cycle. Immunofluorescence studies of Dlg1-depleted Jurkat T cells show that while Dlg1 depletion affects IS formation, it does not affect HIV-1-induced VS formation. Co-culture assays and quantitative cell-to-cell HIV-1 transfer analyses show that Dlg1 depletion does not modify transfer of HIV-1 material from infected to target T cells, or HIV-1 transmission leading to productive infection via cell contact. Dlg1 depletion results in increased virus yield and infectivity of the viral particles produced. Particles with increased infectivity present an increase in their cholesterol content and during the first hours of T cell infection these particles induce higher accumulation of total HIV-1 DNA.

**Conclusion:**

Despite its role in the IS formation, Dlg1 does not affect the VS and cell-to-cell spread of HIV-1, but plays a role in HIV-1 cell-free virus transmission. We propose that the effect of Dlg1 on HIV-1 infectivity is at the stage of virus entry.

## Introduction

Retrovirus spread depends on the correct assembly, budding and transmission of viral particles both by cell-free viral particles and by virus cell-to-cell transfer. *Human immunodeficiency virus type-1* (HIV-1) cell-to-cell virus transmission is known to be more efficient than cell-free virus transmission [1], [2], [3], [4] and the former mode of transmission is probably predominant *in vivo* between cells close to each other, in tissues where primary infection occurs. Cell-to-cell viral transmission is mediated by cellular structures that allow the movement of HIV-1 between cells, such as membrane nanotubes [5], filopods [6] and the stable macromolecular adhesive contact known as virological synapse (VS). The VS forms a tight cleft between an infected cell and a target cell [7], [8], [9], [10], [11] and appears to be the dominant structure involved in cell-to-cell spread of HIV-1 in T cells [12], [13]. VS formation is initiated by the interaction between the viral envelope glycoprotein Env on the surface of the infected cell and the cellular receptor CD4 and the co-receptors CXCR4 or CCR5 on the target cell. This cellular conjugate is stabilized by the adhesion molecule lymphocyte function-associated antigen 1 (LFA-1) together with talin and actin on the target cell [7], [8] and the intracellular adhesion molecule 1 (ICAM-1), which interacts with LFA-1, on the infected cell [13]. The VS shares features and components with the immunological synapse (IS), the cellular conjugate formed between a T cell and an antigen-presenting cell (APC). The IS is formed by recognition between the T cell receptor (TCR) on the T cell and the cognate peptide-major histocompatibility complex (pMHC) on the APC. Importantly, it was reported that HIV-1 infection impairs the formation of the IS [14].

Maximum efficiency of virus assembly, budding and transmission of HIV-1 depend on the host cell machinery recruited by the viral protein Gag that interacts with numerous host proteins, complexes and structures [15], [16], [17]. We previously identified the human homologue of *Drosophila* Discs Large (Dlg1) protein as a new cellular partner of HIV-1 Gag and described Dlg1 as a negative regulator of virus particle infectivity [18]. In Dlg1-depleted cells, Gag production and maturation or virus release were not affected, whereas the viruses produced were fivefold more infectious [18].

Dlg1 is a membrane-associated guanylate kinase (MAGUK), it is an important adaptor protein involved in the assembly of protein complexes at sites of cell-to-cell contact. Dlg1 is a modular protein consisting of a proline-rich N-terminal region, multiple PDZ (PSD-95–DLG–ZO-1) domains, a Src homology 3 domain, a HOOK (protein 4.1 binding) domain and a GUK-like domain. Dlg1 is a scaffolding protein recruited beneath the plasma membrane at cellular contacts such as synapses, adherent junctions and tight junctions, where it plays a key role in clustering protein complexes. It is implicated in T cell signaling, polarity [19], morphology and migration, and in IS formation [20]. In T cells, Dlg1 interacts with the cellular surface proteins, PTA-1, Kv1.3, CD2, and with LFA-1 and it localizes transiently in the IS upon antigen presentation [21]. Dlg1 recruited to the IS interacts with key components of T cell signaling [20], [22], [23], it may link the TCR signaling machinery with regulators of the cytoskeleton. Dlg1 is thought to be involved in coordinating T cell activation and actin polymerization induced by the lymphocyte-specific protein tyrosine kinase (Lck) and mediated by the Wiskott–Aldrich syndrome protein, required for synaptic lipid raft and TCR clustering [24]. In Jurkat T cells, Dlg1 was reported to be an activation antagonist that would promote receptor internalization after initial TCR engagement [21]. It was also shown in Jurkat T cells that Dlg1 and ezrin cooperate to control IS architecture and T cell activation [25]. In addition, it was reported that Dlg1 interacts with the Zeta-chain-associated protein kinase 70 kDa (Zap70) involved in the IS [26], [27] and that Zap70 in donor HIV-1-infected cells was involved in HIV-1 cell-to-cell transmission [28].

Although it is clear that Dlg1 plays a key role in T cells by organizing signaling complexes at the IS, its role in the VS or in cell-to-cell virus transmission has not been studied. Given these key roles of Dlg1, we expected that it could affect VS formation and/or HIV-1 cell-to-cell transmission. In this study the possible implication of Dlg1 in HIV-1 infection of a natural target of the virus was investigated in T cells depleted of Dlg1. We demonstrate for the first time that despite the key role of Dlg1 in the IS formation and T cell signaling, Dlg1 does not affect VS formation or cell-to-cell spread of HIV-1. Our results show a specific role of Dlg1 in HIV-1 cell-free virus transmission and allow us to postulate that by modifying viral cholesterol content Dlg1 affects the step of virus-cell fusion.

## Materials and Methods

### Cells and cell culture

CD4+/CXCR4+ Jurkat T cells (clone 20; a kind gift of Dr. Olivier Schwatz, Institut Pasteur, Paris, France), APC (Raji B cells; a kind gift of Dr. Georges Bismuth, Institut Cochin, Paris, France) and Jurkat LTR-luciferase T cells (1G5 cells; AIDS Research and Reference Reagent Program) were maintained in complete RPMI medium: RPMI 1640 (Gibco) supplemented with 10% FCS, streptomycin (100 µg/mL; Gibco), penicillin (100 U/mL; Gibco), glucose (0.43%, Gibco) and glutamine (2 mM; Gibco). Peripheral blood mononuclear cells (PBMCs) were isolated from whole blood or buffy coats (Établissement Français du Sang, St Vincent de Paul, Paris, France) by Ficoll-Hypaque (Sigma) gradient centrifugation. Primary CD4+ T cells were purified by negative selection with a CD4+ T cell enrichment kit (Human CD4 T lymphocyte enrichment set-DM, BD Biosciences) according to the manufacturer's protocol. The primary CD4+ T cells were first activated with phytohemaglutinin A (PHA-L; 3.0 µg/mL; Sigma-Aldrich) and interleukin-2 (IL2; 50 IU/mL; PeproTech) then maintained in culture with IL2 (50 IU/mL). 293T and HeLa P4.2 cells (Hela-CD4-HIV-LTR-lacZ cells) were maintained in DMEM medium (Gibco) supplemented with 10% FCS, streptomycin (100 µg/mL), penicillin (100 U/mL) and glutamine (2 mM).

### Antibodies

Primary antibodies used to detect the HIV-1 envelope protein on immunoblots were the anti-gp120 goat polyclonal (1/10000; Abcam) and the monoclonal antibody (mAb) anti-Env 5F7 (1/200; AIDS Research and Reference Reagent Program) [29]. Other primary antibodies used on immunoblots were: the HIV-1 anti-CA-p24 #24-2 mAb for HIV-1 Gag (1/5000; AIDS Research and Reference Reagent Program), the anti-tubulin mAb (1/5000; Sigma-Aldrich) for tubulin, the anti-GAPDH 6C5 mAb (1/1000; Santa Cruz Biotechnology) for GAPDH. The anti-PSD-95 mAb (PDZ domain; US Biological) was used for detection of Dlg1 on immunoblots (1/2500) and by immunofluorescence (IF; 1/100). Other primary antibodies used for IF were the HIV-1_SFE_ p24 rabbit antiserum (1/5000; AIDS Research and Reference Reagent Program) for Gag, the anti-CD4 mAb MAB379 (1/50; R&D systems) for the CD4 receptor. The anti-pericentrin rabbit polyclonal antibodies (1/2500; Abcam) were used to visualize the microtubule organizing center (MTOC) by IF and the 4G10 mAb (1/2; Ubi) for phosphotyrosine. Primary antibodies were detected with Cy3-, Cy5-, Alexa 568-, or Alexa 647-conjugated goat anti-mouse (1/500; Invitrogen), goat anti-rabbit (1/500; Invitrogen), donkey anti-mouse (1/2000; Invitrogen), or donkey anti-rabbit (1/2000; Invitrogen) secondary antibodies.

The anti-CD3-phycoerythrin (PE; 1/100; BD Pharmingen), anti-LFA-1 (1/20; Thermo Scientific), anti-CD4-PE (1/20; Beckman Coulter), anti-Human CD54-biotin (anti ICAM-1; 1/100; eBioscience), anti-CXCR4 12G5 mAb (1/100; AIDS Research and Reference Reagent Program) [30] and anti-Env 5F7 (1/20) were used to detect CD3, LFA-1, CD4, ICAM-1, CXCR4 and HIV-1 Env respectively by flow cytometry. PE-conjugated secondary antibodies (1/200; Dako) or PE-conjugated streptavidin (1/1000; eBioscience) were used when necessary. For intracellular Gag measurements the anti-HIV-p24 KC57-PE mAb (1/500; Coulter Beckman) was used.

### Plasmids

The HIV-1 proviral clones pNL4-3 and LAI.2 were obtained from the NIH AIDS Research and Reference Reagent Program. The luciferase-expressing vector pNLuc [31] and the envelope protein expression vector pIIINL4env [32] were kind gifts from Dr. Eric Freed (NIH). The control siRNA [33] and the siRNA directed to the N-terminal domain specific for Dlg1 (nucleotides 569 to 587: 5′UGAAGUGAUAGGUCCAGAA3′; accession no. NM_004087) were obtained from QIAGEN (Courtaboeuf, France). The lentiviral vector pHIV-H1shRNADlg1 (pV2.3.60; generated by Vectalys, Labège, France) encodes the GFP gene and a short hairpin RNA (shRNA) directed against Dlg1 for its depletion (same sequence as for siRNA, nucleotides 569 to 587). The siRNA and shRNA specifically target the seven known isoforms (splicing variants; http://www.uniprot.org/uniprot/Q12959#section_comments) of human Dlg1, but not the other members of the Dlg family of MAGUKs [34]. The control lentiviral vector pshRNACtl encodes the GFP gene and an irrelevant shRNA control sequence. The lentiviral vector Mission shRNADlg1 (NM_004087; Sigma) encodes the puromycin resistance gene and an shRNA sequence for Dlg1 depletion (5′CCGGGCACAGATGCAGATTATGAATCTCGAGATTCATAATCTGCATCTGTGCTTTTT3′). The control lentiviral vector Mission shRNACtl encodes the puromycin resistance gene and an irrelevant shRNA sequence.

### Transduction, transfection and infection

Jurkat T cells were transduced with one of the lentiviral vectors in the presence of 10 mM Hepes and 2 µg/mL DEAE to improve transduction. The stable Dlg1-depleted T cell lines were obtained using the following lentivirus-based vectors. The GFP-encoding lentiviral vectors pHIV-H1shRNADlg1 and pshRNACtl were used to obtain stably transduced Jurkat cells depleted of Dlg1 (referred to as Dlg1-) or control cell lines expressing endogenous Dlg1 (referred to as Dlg1+) respectively. After 3 h incubation at 37°C, complete fresh RPMI medium was added. Cells transduced with the GFP-encoding pHIV-H1shRNADlg1 or shRNACtl vectors were cultured for at least a week at 37°C before sorting the Dlg+ and Dlg1- cells for GFP positive cells by flow cytometry. Cells transduced with the puromycin-encoding Mission shRNA vectors were cultured for three days without selection and thereafter in RPMI supplemented with puromycin (0.75 mM/mL; Gibco).

The X4 NL4.3 and LAI.2 strains of HIV-1 were produced in 293T cells (1.5×10^6^) transfected with 5 µg of pNL4.3 or LAI.2 proviral plasmids by the calcium phosphate technique and supernatants of cultured cells were collected 48 to 72 h post-transfection. For the production of HIV-1 in 293T cells depleted of Dlg1 by siRNA, two successive transfections were performed on 1.5×10^6^ cells. The first transfection was with 20 nM siRNA and the second one 24 h later, with the same amount of siRNA together with 5 µg of LAI.2 proviral plasmid. Viruses were then filtered through a 0.45 µm pore-size filter and assayed for p24 content with the ELISA INNOTEST HIV Antigen mAb (INGEN). T cell lines were infected with HIV-1 NL4.3 at a multiplicity of infection (MOI) of 0.005 to 0.05 in small volumes (100 to 500 µL) of RPMI medium containing 10 mM Hepes and 2 µg/mL DEAE to improve viral adhesion. RPMI alone was used for infections in which the properties of viral particles produced by Dlg1+ or Dlg1- cells were compared. After 2 h incubation at 37°C the viral inoculum was washed off with complete RPMI medium and the cells were maintained in culture at 37°C.

Kinetics of infection were followed by determining the fraction of HIV-1-infected cells in the T cell cultures measuring intracellular Gag by flow cytometry. Cells were fixed, permeabilized (as described for immunofluorescence) and stained with the anti-HIV-p24 KC57-PE mAb which recognizes the 55, 39, 33 and 24 kDa proteins of the core of HIV-1.

Single cycle pseudotyped virions were produced in 293T cells by co-transfecting, as described above, 5 µg of the luciferase-expressing vector pNLuc with 0.5, 0.75 or 1 µg of the HIV-1 envelope-expressing vector pIIINL4env or with 50 ng of the plasmid encoding the *Vesicular stomatitis virus* glycoprotein G (VSV-G). The pseudotyped virions were normalized for p24 and used to infect Dlg1 + or Dlg1- Jurkat T cells. A range of viral input from 100 to 1000 ng of p24 was used to infect Dlg1+ and Dlg1- Jurkat T cells as described above. Two dpi, the cells were lysed and luciferase activity was determined.

### Quantification of HIV-1 total DNA

HIV-1 NL4.3 and LAI.2 viral stocks produced by cells expressing or not Dlg1 were treated with 3 IU of Dnase I (Qiagen) per µg of p24 for 1 h at 37°C before infection of 2×10^6^ Jurkat cells in RPMI as described above. Cells were harvested 6 h later, washed three times in PBS (Gibco) and DNA was extracted using the QIAamp Blood DNA minikit (Qiagen). Quantification of viral DNA was performed in triplicate by quantitative PCR on a Light Cycler 480 instrument (Roche Diagnostics, Meylan, France) using the Light Cycler 480 probe master (Roche Diagnostics) and the previously described HIV-1-specific primers and fluorogenic probes [35]. The forward primer MH531 anneals to the U5 region of the LTR (5′TGTGTGCCCGTCTGTTGTGT3′), the reverse primer MH532-NL4.3 anneals to the upstream region of *gag* (5′GAGTCCTGCGTCGAGAGATC3′) and the fluorogenic probes for these region are MHFL: 5′CCCTCAGACCCTTTTAGTCAGTGTGGAA-fluorescein and MHLC: 5′ LC640TCTCTAGCAGTGGCGCCCGAACAG-PH. After an initial denaturation step (95°C for 8 min), 40 cycles consisting of 95°C for 10 s, 60°C for 10 s and 72°C for 6 s were performed. The second-derivative-maximum method provided by the Light Cycler SW 1.5 quantification software was used (Roche Diagnostics). The reference gene was human ß-globin, amplified using the Light Cycler control kit DNA (Roche Diagnostics) that includes human genomic DNA and ß-globin-specific primers and probes. Viral DNA quantification was determined using the ΔCq method [36]. For each HIV-1 DNA and globin, the mean Cq of all samples was used to calculate the ΔCq. The calculations performed for all the values were: the ΔCq  =  the mean Cq – each Cq, the RQ  =  Efficiency of gene's amplification^ΔCq^ and the normalized NRQ  =  RQ HIV-1 DNA/RQ globin.

### Infectivity tests and particle analysis

Supernatants of Dlg1+ and Dlg1- Jurkat T cells infected with HIV NL4.3 at a multiplicity of infection (MOI) of 0.05 were collected 7 days post-infection (dpi). Equal amounts of virus (from 1 to 5 ng) determined either by p24 content or by reverse transcriptase (RT) activity (quantified by the Quan-T-RT assay system, Amersham Bioscience) were used to infect HeLa P4.2 reporter cells. After 36 h of incubation, the cells were lysed and β-galactosidase production was assessed by a colorimetric assay based on cleavage of chlorophenol red-ß-D-galactopyranoside (CPRG) as described previously [18]. The mean optical density value of four experiments obtained from extracts of cells infected with viruses from Dlg1+ Jurkat T cells was taken as 100%.

Single-cycle infectivity analyses were performed by using HIV-1 pseudotypes obtained with the pNLuc [31] vector together with the HIV-1 Env expression vector pIIINL4env [32] to determine the efficiency of early steps of the HIV-1 life cycle in cells expressing or not Dlg1. Cells were lysed 48 h post-infection and luciferase activity was measured using the luciferase assay reporter kit (Promega) according to the manufacturer's instructions.

Purified virions were obtained from supernatants of infected cultures, filtered (0.45 µm) and ultracentrifuged through a 25% sucrose cushion in TNE buffer (100 mM NaCl, 10 mM Tris-HCl, pH 7.4, and 1 mM EDTA). Ultracentrifugation was performed at 150000 X g for 1 h at 4°C in a Beckman SW41 Ti rotor and viral pellets were resuspended in 30 µL lysis buffer (20 mM Tris-HCl pH 8, 0.2 mM EGTA, 120 mM NaCl, 0.2 mM NaF, 0.2% Sodium deoxycholate, 0.5% NP40, and complete protease inhibitors; Roche Applied Science) before immunoblotting.

### Determination of viral cholesterol content

HIV-1 LAI.2 virions were recovered, ultracentrifuged and solubilized as described above, and were normalized according to their p24 content to determine their cholesterol content using the Amplex Red Cholesterol Assay Kit (Invitrogen) according to the manufacturer's protocol. The media from uninfected cells were prepared the same way as the virions and were used as controls in the cholesterol assay to evaluate contaminating cholesterol not associated to virions.

### Co-culture assays

To quantify HIV-1 transfer from infected to target cells an adaptation of the co-culture assay reported by Sourisseau *et al.* [2] was used. Dlg1+ or Dlg1- Jurkat T cells were infected with HIV-1 NL4.3 at an MOI of 0.005 or 0.05. Once the cultures had reached a minimum of 20% infection, infected (Gag+/GFP+) cells were co-cultivated at a 1∶1 ratio for up to 17 h with target uninfected Jurkat cells (GFP-) or with primary CD4+ T cells stained with cell trace violet (Invitrogen). Samples were collected and analyzed for Gag transfer into target cells at different time points of the co-culture by intracellular Gag staining with KC57-PE mAb followed by flow cytometry analysis (Canto 2 cytometer or FC-500 Cytomics).

This co-culture test was also adapted to measure productive HIV-1 transmission into the target cells. Dlg1+ or Dlg1- Jurkat T cells infected as described above were co-cultivated at a ratio of 1∶1 with uninfected Jurkat LTR-luciferase cells (1G5 cells) for 24 h. Luciferase activity was then measured in target 1G5 cells after lysis using the Promega luciferase kit, according to the manufacturer's instructions.

### Immunofluorescence and laser scanning confocal microscopy

HIV-1-infected Dlg1+ and Dlg1- Jurkat T cells, 5 to 7 dpi when cultures showed at least 50% Gag positive cells were allowed to form contacts. Infected cells (500 000 cells) were mixed at a 1∶1 ratio with target uninfected primary CD4+ T cells and deposited for an hour at 37°C on poly-L-Lysine-coated coverslips to allow contact formation. For intracellular staining, cells were fixed with 4% paraformaldehyde, permeabilized with methanol and washed with PBS buffer containing 2% BSA and 0.1% Tween 20. The cells were fixed and permeabilized to label internal Gag, Dlg1, and CD4 (with the blocking antibody MAB379) after VS formation. The cells were then incubated 40 min at room temperature with the primary antibodies anti-p24, anti-PSD-95 or anti-CD4 diluted in PBS buffer containing 2% BSA and 0.1% Tween 20. Primary antibodies were detected with Cy3-, or Cy5-conjugated goat anti-mouse, and anti-rabbit secondary antibodies. Monosynapses were defined as single cell-to-cell contacts presenting enrichment of Gag on the infected cell and co-localization with CD4 on the CD4+ target cell at sites of contact. Polysynapses were defined as contacts between a single effector cell and multiple target cells.

For the visualization of MTOC polarization, IS conjugates were formed between Jurkat and APC (Raji B) cells. Phosphotyrosine accumulation at the interface between the two cells was used as a marker of a productive IS contact that has led to TCR activation and to activation of the downstream signaling cascade. Centrin was used simultaneously to localize the MTOC. APCs were pulsed with 200 ng/mL superantigen in serum-free RPMI 1640 and plated on coverslips for 30 min at 37°C. Superantigen was a mix of recombinant staphylococcal enterotoxin E, A, B, and C3 (Toxin Technology). Dlg1+ or Dlg1- Jurkat cells were loaded with Hoechst and added to the APCs for 14 min at 37°C to allow conjugate formation. Cells were fixed with 2% paraformaldehyde for 10 min at room temperature and washed with PBS containing 0.5% BSA and 0.1% saponin. The cells were then incubated with the primary antibodies anti-pericentrin and anti-phosphotyrosine for 30 min. Primary antibodies were detected with Alexa 568-, or Alexa 647-conjugated donkey anti-mouse or donkey anti-rabbit secondary antibodies.

Coverslips were mounted with Fluorsave reagent (Calbiochem) or the ProLong anti-fade kit solution (Invitrogen). Laser scanning microscopy was performed using a Leica TCS resonant scanner multi-photon (spinning disc) or a Leica TCS SP2 AOBS, with the 63X objectives. Epifluorescence images were acquired with a Nikon TE2000, a CCD 16 Bits camera (Cascade, Princeton Instruments) and the Metamorph software (Molecular Devices). Images were analyzed using ImageJ software (Wayne Rasband; NIH). For quantitative image analysis of MTOC polarization at the IS, a conjugate was defined as a contact between a Jurkat and an APC with phosphotyrosine accumulation at the IS. The ratio between the distance from the MTOC to the IS and the cell diameter were calculated. The MTOC was considered relocalized if the ratio was inferior to the threshold value of 20%.

### Electron Microscopy

Dlg1+ and Dlg1- HIV-1-infected Jurkat cells (3.10^6^) six or seven days post-infection, when the cultures showed at least 60% Gag+ cells, were fixed for 4 h in a solution containing 2% paraformaldehyde, 2.5% glutaraldehyde and 2.5% sucrose. The cells were washed in PBS and centrifuged at 20000 X g. Subsequently, the cellular pellet was washed three times in 0.1 M sodium phosphate buffer pH 7.0, incubated 30 min at 4°C in osmic acid (1% in sodium phosphate buffer) and dehydrated in graded ethanol solutions (2x10 min in 70%, 2x10 min in 90% and 2x10 min in 100% ethanol). The cell pellet was then incubated 2x1 min in propylene oxide, 10 min in 50% Epon-50% propylene oxide, then embedded in Epon and incubated for polymerization 24 h at 60°C. Finally, 90 nm sections were cut on a Reichert Ultracut S and layed on a grid for 10 min of staining with 2% uranyl acetate and 4 min with lead citrate at room temperature. The observations were performed with a JEOL 1011 transmission electron microscope and the images recorded at 80 kv with a GATAN Erlangshen camera 1000. For quantifications a total of 5300 Dlg1+ Jurkat T cells and of 6500 Dlg1- Jurkat T cells were observed.

### Statistical analysis

Statistical analyses were performed using the Mann Whitney and chi^2^ tests. Statistical significance was defined for P values <0.05.

## Results

### Kinetics of HIV-1 replication in Dlg1-depleted T cells

**Figure 1:**
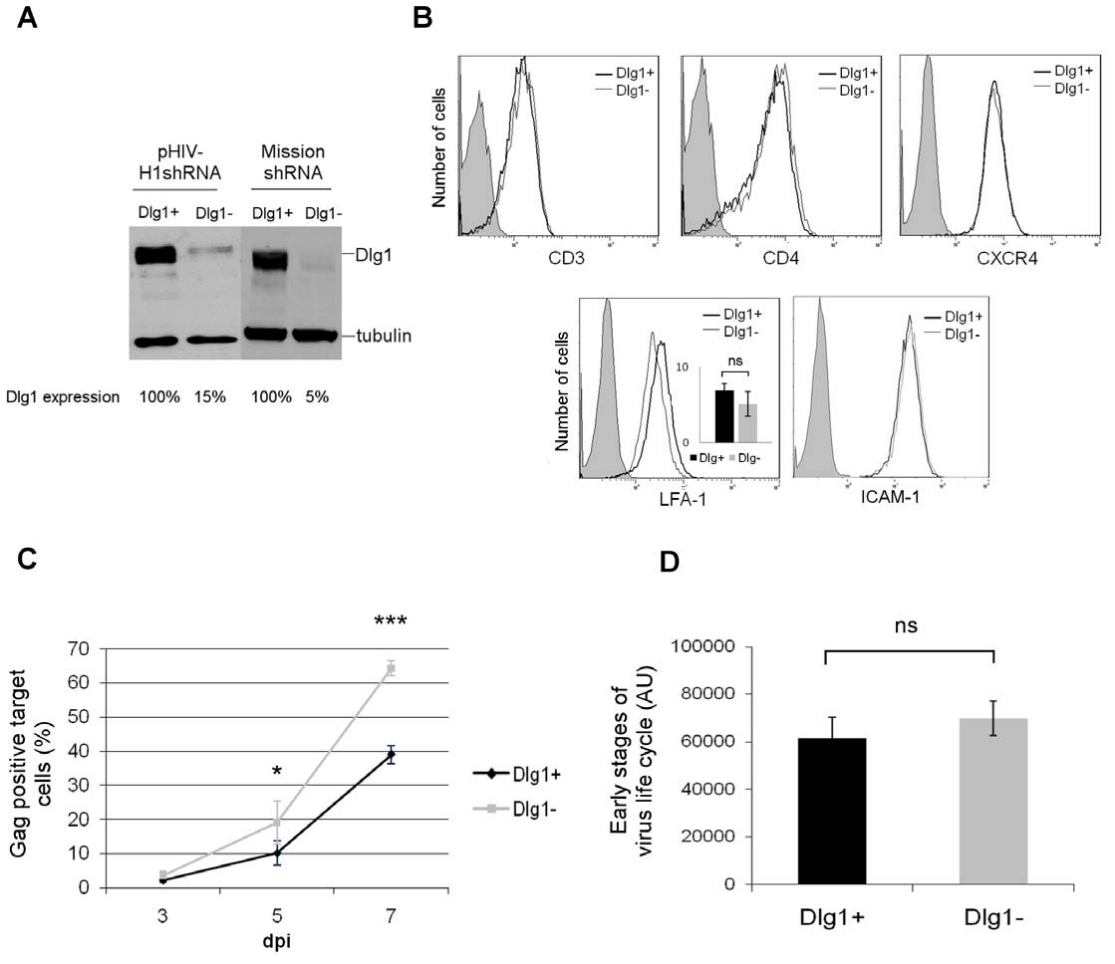
HIV-1 replication is enhanced in Dlg1-depleted T cells. **A. Dlg1 expression in Dlg1+ and Dlg1- Jurkat T cells obtained with two lentivirus-based shRNA vectors.** The stable Dlg1-depleted Jurkat T cell lines (Dlg1-) were obtained using two lentivirus-based vectors that target two different sequences on Dlg1. Control Jurkat T cell lines (Dlg1+) were obtained using the control vectors. T cells transduced with the GFP-encoding vectors (pHIV-H1shRNADlg1 or pHIV-H1shRNACtl) or with the puromycin-encoding vectors (Mission shRNADlg1 or Mission shRNA Ctl) were analyzed by western blot, and Dlg1 levels were quantified using ImageJ software. **B. Expression of surface molecules in Dlg1+ and Dlg1- Jurkat T cells.** Cells were labeled with antibodies against CD3, CD4, CXCR4, LFA-1 and ICAM-1 and analyzed by flow cytometry. The data are representative of three independent experiments performed in triplicates. For LFA-1 the mean values of three independent experiments performed in triplicates is also presented. **C. HIV-1 replication in Dlg1+ and Dlg1- Jurkat T cells infected with the NL4.3 HIV strain.** Viral replication was measured by determining the fraction of HIV-1-infected cells in the two cultures by intracellular Gag labeling and flow cytometry. Cells were labeled with the anti-HIV-p24 mAb KC57-PE, 3, 5 and 7 dpi. The data are the means of four independent experiments performed in duplicate. P = 0.031 for 5 dpi. P<0.0001 for 7 dpi. **D. Efficiency of early steps of the HIV-1 life cycle in cells expressing or not Dlg1.** Dlg1+ and Dlg1- Jurkat T cells were infected with the single cycle pNL4.3-derived pNluc vector pseudotyped with the HIV-1 Env expression vector pIIINL4env and luciferase activity was measured 48 h post infection. The data are the means of three independent experiments performed in triplicate, using 100 ng to 1000 ng of p24 of virus produced by 293T cells co-transfected with 5 µg of pNLuc and 0.5, 0.75 or 1 µg of pIIINL4env. P = 0.59. Error bars represent standard error of the mean (SEM). ns  =  no statistically significant difference. *, P<0.05. ***, P<0.001. AU  =  arbitrary units.

To establish an experimental system that allows kinetic studies of HIV-1 infection in a natural host cell of the virus, Jurkat T cells were stably depleted of Dlg1 and infected by HIV-1. This system is physiologically more relevant than the one used previously with adherent 293T cells depleted of Dlg1 by siRNA and transfected with a proviral HIV-1 plasmid [18]. The stable Dlg1-depleted Jurkat T cell lines (Dlg1-) were obtained using two lentivirus-based vectors that target two different sequences in Dlg1 indicated in the materials and methods section. The depletion of Dlg1 obtained in Dlg1- Jurkat T cells using the GFP-encoding pHIV-H1shRNA lentivirus was 85% compared to Dlg1 control (Dlg1+) Jurkat T cell levels (Figure 1A). The depletion of Dlg1 obtained using the puromycin-encoding Mission shRNADlg1 lentivirus in Dlg1- Jurkat T cells was 95% compared to control Dlg1+ Jurkat T cell levels (Figure 1A).

Given the key role of Dlg1 in clustering protein complexes at the plasma membrane, in T cell signaling and in IS formation, the Dlg1- T cells were characterized to determine if the absence of Dlg1 affects the expression of surface molecules that are particularly important for T cell function and for HIV infection. The surface level of receptors that are components of the IS (CD3, LFA-1, ICAM-1), or the VS (CD4, LFA-1) and the viral receptor/co-receptor (CD4, CXCR4) were determined by flow cytometry after staining with specific antibodies. The surface levels of CD3, CD4, CXCR4, LFA-1 and ICAM-1 were similar in Dlg1- and Dlg1+ cells (Figure 1B), indicating that Dlg1 knock down does not have a general deleterious effect.

The course of HIV-1 replication during infection was followed in Dlg1+ and Dlg1- Jurkat T cells infected with the NL4.3 HIV strain by measuring intracellular Gag by flow cytometry. The propagation of the virus was more efficient in Dlg1- cells, with 40% more infected cells seven days-post infection (dpi), indicating that Dlg1 affects the replication of HIV-1 in T cells (Figure 1C).

We wondered whether the increase in HIV-1 replication was due to an effect of Dlg1 in either early or late steps of the virus life cycle. To this end, the efficiency of early steps of the HIV-1 life cycle in cells expressing or not Dlg1 was determined by using single-cycle HIV-1 infectivity assays in the absence of virus spread. The pNL4.3-derived pNluc vector, which is Envelope and Nef minus and expresses luciferase following infection [31], together with the HIV-1 Env expression vector pIIINL4env [32] were used to produce pseudotyped viruses. Within the range of viral inputs tested from 100 to 1000 ng of p24, no difference in the levels of luciferase activity was observed between cells expressing or not Dlg1 (Figure 1D). These results suggest that Dlg1 does not interfere with early steps of HIV-1 life cycle, therefore it may affect late steps of the virus life cycle such as virus assembly, budding, cell-free virus transmission or cell-to-cell transmission.

### Virological synapse formation in Dlg1-depleted T cells

**Figure 2:**
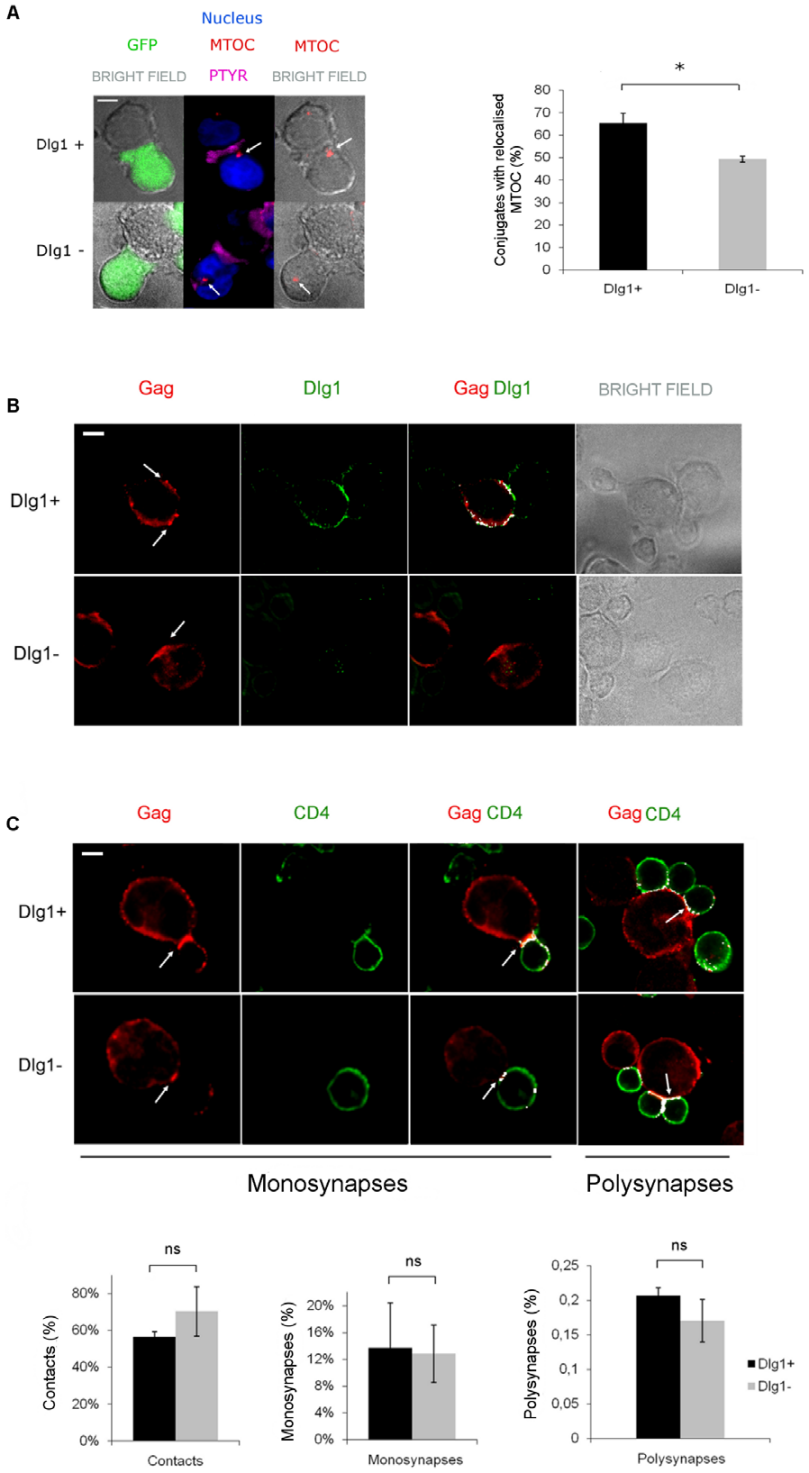
Dlg1 depletion affects IS but not VS formation in T cells. **A. MTOC polarization toward the nascent IS in Dlg1+ and Dlg1- Jurkat T cells.** Left panel: IS conjugates were formed between Jurkat and APC (Raji B) cells. Phosphotyrosine accumulation at the interface between two contacting cells served as marker of a productive IS contact that led to TCR activation and to activation of the downstream signaling cascade. Centrin was visualized simultaneously to localize the MTOC. A polarized MTOC in a Dlg1+ T cell and an unpolarized MTOC in a Dlg1- T cell are seen. The nuclei of Jurkat T cells are shown in blue, the MTOCs are shown in red and indicated by a white arrow. The phosphotyrosine (PTYR) is shown in magenta. The results shown were obtained with the pHIV-H1shRNADlg1 and pHIV-H1shRNACtl vectors expressing the GFP. Similar results were obtained with cells transduced with the lentivirus vectors pHIV-H1shRNADlg1 and Mission shRNADlg1. Right panel: **Quantification of MTOC polarization** observed in Dlg1+ (n = 131) and Dlg1- (n = 151) T cells in four independent experiments. P = 0.029. **B. Cellular localization of Gag in the presence and absence of Dlg1.** Dlg1+ and Dlg1- Jurkat T cells were immunostained for Gag and for Dlg1. White arrows indicate Gag accumulation at the VS. The images shown are representative of three independent experiments. **C. VS formation in Dlg1+ and Dlg1- Jurkat T cells.** Upper panel: HIV-1-infected Dlg1+ and Dlg1- Jurkat T cells were mixed with an equal number of non-infected primary CD4+ target cells and were allowed to establish contacts on poly-L-lysine-coated coverslips for 1 h at 37°C. The cells were fixed, permeabilized, immunostained for Gag and CD4 and analyzed by confocal microscopy. Monosynapses were defined as contacts between a single effector and a single target cell presenting co-localization (in white) of Gag on the infected cell and of CD4 on the CD4+ target cell at sites of contact. Polysynapses were defined as contacts between a single effector cell and multiple target cells. Lower panel: Quantification of cellular contacts, monosynapses and polysynapses between HIV-1-infected Dlg1+ or Dlg1- Jurkat T cells and CD4+ target cells. A total of 1603 Dlg1+ control T cells and 1616 Dlg1- T cells were counted. The data are the means of 3 independent experiments. P = 0.7 for contacts. P = 1.0 for monosynapses. P = 1.0 for polysynapses. Error bars represent SEM. ns  =  no statistically significant difference. *, P<0.05. Scale bar  =  5 µm.

The Dlg1- T cells were functionally characterized by looking at the MTOC polarization during IS formation between T cells and APCs. In T cells, MTOC polarization to the plasma membrane modulates IS formation and T cell signaling [37], [38] and Dlg1 silencing reduced MTOC positioning at the IS [25]. Phosphotyrosine accumulation at the interface between the conjugated cells was used as a marker of a productive IS contact that has led to TCR activation and to activation of the downstream signaling cascade. Centrin was visualized simultaneously to localize the MTOC. Figure 2A shows results obtained with the pHIV-H1shRNADlg1 and pHIV-H1shRNACtl vectors expressing the GFP. As expected, MTOC polarization towards the plasma membrane during IS formation was reduced in Dlg1- cells. An example of a polarized MTOC in Dlg1+ cells and of a non-polarized MTOC in Dlg1- cells are depicted (Figure 2A left panel). The quantification of MTOC polarization in Dlg1+ and Dlg1- cells (Figure 2A right panel) shows a statistically significant reduction of MTOC polarization towards the plasma membrane during IS formation in Dlg1- cells. Therefore, these cells can be used to further study the possible role of Dlg1 in VS formation and virus transmission during HIV-1 infection.

To determine the cellular localization of Gag in the presence and absence of Dlg1, HIV-1-infected Dlg1+ and Dlg1- T cells were immunostained for Dlg1 and Gag. Dlg1 is present at the cellular periphery and particularly at cell contacts in Dlg1+ cells and no Dlg1 was observed in Dlg1- cells (Figure 2B). Gag is at the cellular periphery and accumulates at cell contacts between infected and target cells in both Dlg1+ and Dlg1- cells as indicated by white arrows (Figure 2B and C).

Jolly *et al.* [7] described HIV-1-induced VS formation as the coenrichment of the viral Gag and Env proteins on the infected cell and of CD4 on the primary target cell at sites of cell-to-cell contact. Monosynapses were scored here as single cell-to-cell contacts presenting enrichment of Gag on the HIV-1-infected cell and colocalization with CD4 on the target cell at sites of contact. Polysynapses were defined as contacts between a single effector cell and multiple target cells. Using these criteria, virological monosynapses and polysynapses formed between donor Dlg1+ or Dlg1- cells and Jurkat target T cells appeared similar (data not shown). Since the mAb used for these studies is a CD4 blocking mAb, staining of permeabilized CD4+ primary cells allowed the detection of CD4 not bound to gp120 and reduction of CD4 staining in the target cell at the site of contact may be observed. Similar results were obtained for monosynapses and polysynapses formed between donor Dlg1+ or Dlg1- cells and primary CD4+ target T cells (Figure 2C). As indicated by the white arrows, Gag and CD4 colocalized at the VS (Figure 2C top panel). After one hour of co-culture, Gag puncta were visible on the target primary CD4+ cells both on monosynapses and on polysynapses with either Dlg1+ or Dlg1- cells. The quantification of cellular contacts, monosynapses and polysynapses of three independent experiments revealed that Dlg1- T cells were not impaired in HIV-1-induced VS formation (Figure 2C bottom panel).

Together, these results show that in HIV-1-infected T cells the absence of the scaffold protein Dlg1 does not affect Gag localization to VS, cell-to-cell contact or VS formation in both monosynapses and polysynapses.

### HIV-1 cell-to-cell transmission in Dlg1-depleted T cells

**Figure 3:**
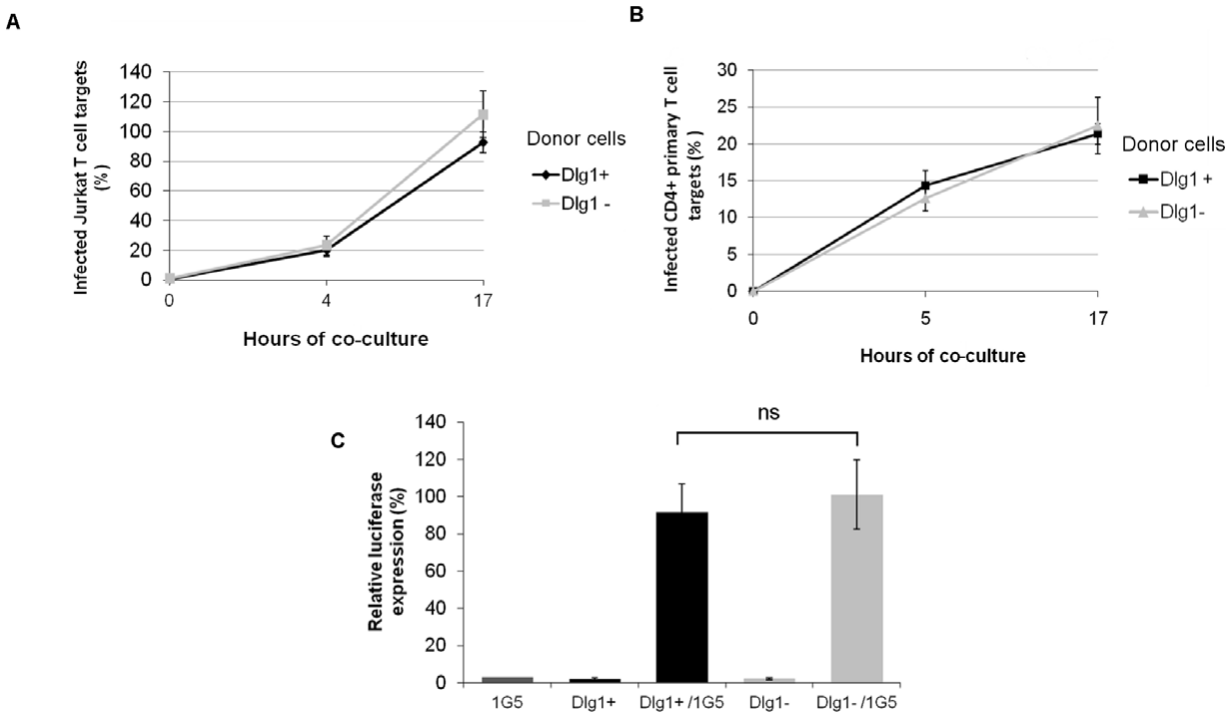
HIV-1 cell-to-cell transmission is not affected in Dlg1-depleted T cells. **A. Co-culture of HIV-1-infected Dlg1+ and Dlg1- Jurkat and target Jurkat Dlg+ T cells.** Dlg1+ and Dlg1- Jurkat T cells infected with HIV-1 NL4.3 were co-cultured with target Jurkat cells. When a minimum level of 20% infection was reached for both Dlg1+ and Dlg1- cells, equal numbers of infected and target cells were mixed and co-cultured for up to 17 h. At different times after infection the cells were analyzed by flow cytometry to determine the percentage of infected target cells. Results are mean relative values of six independent experiments performed in duplicate. For each experiment the values were normalized taking as 100% the value obtained for one of the duplicates of Dlg1+ cells at 17h. P = 0.26 at 17 h. Assays were performed with cells transduced with the two lentivirus vectors (pHIV-H1shRNA and Mission shRNA) and similar results were obtained. The results obtained with the GFP-encoding vectors are presented. **B. Co-culture of HIV-1-infected Dlg1+ and Dlg1- Jurkat and target CD4+ primary T cells.** Dlg1+ and Dlg1- Jurkat T cells infected with HIV-1 NL4.3 were co-cultured with primary CD4+ T cells previously labeled with cell trace violet. Infected and target cells were co-cultured at a 1∶1 ratio for up to 17 h and analyzed by flow cytometry. The data are average values of three independent experiments performed in duplicate. P = 0.31. **C.** **Co-culture of HIV-1-infected Dlg1+ and Dlg1- Jurkat and target Jurkat-LTR-luciferase T cells.** Dlg1+ and Dlg1- Jurkat T cells infected with HIV-1 NL4.3 were co-cultured with Jurkat-LTR-luciferase cells (1G5 cells). Infected and target cells were co-cultured at a 1∶1 ratio for up to 24 h and analyzed for luciferase activity. The data are average relative values of three independent experiments performed in duplicate. The values were normalized taking the values obtained for 1G5 cells at 24 h as background for each experiment. P = 0.7. Error bars represent SEM. ns  =  no statistically significant difference.

We further studied whether Dlg1 could affect HIV-1 cell-to-cell transmission. It has been largely documented that due to faster transmission kinetics of cell-to-cell spread than cell-free virus transmission, 24 h of co-culture is the time frame in which spread of HIV-1 occurs by direct cell-to-cell transmission rather than by free virus transmission [1], [2], [39], [40]. Here, an equal number of infected cells and target cells was mixed and co-cultured for up to 17 h. Virus transfer from infected Dlg1+ and Dlg1- Jurkat T cells to target Jurkat or primary CD4+ T cells was quantified by measuring intracellular Gag in target cells by flow cytometry at different times. Gag transfer from Jurkat donor to Jurkat target T cells was detected within 4 h of co-culture, increasing approximately fivefold by 17 h. At 17 h of co-culture no significant difference was observed in the number of infected Jurkat T cell targets, whether the donor cells were Dlg1+ or Dlg1- Jurkat T cells (Figure 3A). Similar assays were performed with HIV-1-infected Jurkat donor T cells and Dlg1+ or Dlg1- Jurkat targets T cells and no difference was found in the number of infected Jurkat T cell targets, whether the target cells were Dlg1+ or Dlg1- Jurkat T cells (data not shown).

Co-culture assays were also performed with cell trace violet-labeled primary CD4+ T cells as targets and no difference was found in the number of primary CD4+ target T cells infected whether the donor cells were Dlg1+ or Dlg1- Jurkat T cells (Figure 3B).

To determine the level of viral transfer that results in effective viral transmission, Jurkat-LTR-luciferase cells (1G5 cells) were used as target cells. This cell line allows the quantification of effective viral transmission by measuring the luciferase activity. Similar luciferase activity was observed in 1G5 cells after co-culture whether the donor infected cells were Dlg1+ or Dlg1- Jurkat T cells (Figure 3C).

To evaluate the contribution of cell-free virus transmission during the co-culture, supernatants of the different co-cultures were further incubated under the same experimental conditions for an additional 24 h period with target cells either primary CD4+, Jurkat or 1G5 cells. No infection of target cells was detected (not shown), confirming that our experimental conditions allowed us to study cell-to-cell virus transmission. The results of the different co-culture assays show that Dlg1 does not affect HIV-1 cell-to-cell transfer or effective virus transmission in T cell lines or primary T cells. Taken together, our data indicate that the replication enhancement observed in Dlg1- T cells (Figure 1C) is not the result of Dlg1 effects in cell-to-cell contact, VS formation or cell-to-cell spread of HIV-1.

### Levels of HIV-1 Gag and Env proteins in Dlg1-depleted T cells and in particles produced

**Figure 4:**
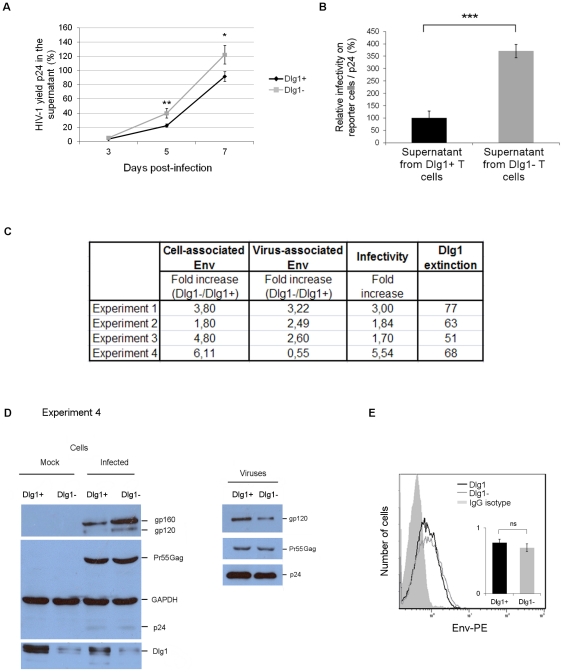
The levels of HIV-1 Env incorporated into viral particles produced by Dlg1-depleted T cells do not correlate with increased infectivity. **A. HIV-1 yield of Dlg1+ and Dlg1- T cells.** Supernatants were collected during the course of infection, filtered and the p24 content was measured by ELISA. The values were normalized for protein content of extracts of cultured cells. The data are the mean values of three independent experiments each carried out in duplicate. For each experiment the values were normalized taking as 100% the value obtained for one of the duplicates of Dlg1+ cells at 7 dpi. P = 0.007 for 5 dpi. P = 0.02 for 7 dpi. **B. Infectivity of HIV-1 particles produced by Dlg1+ and Dlg1- T cells.** Viral supernatants of Dlg1+ and Dlg1- Jurkat T cells infected with HIV-1 NL4.3 were collected 7 dpi, filtered and used to infect indicator HeLa P4.2 reporter cells. Equal amounts of virus determined by p24 quantification were used. ß-galactosidase production was assessed by a colorimetric assay based on cleavage of CPRG. The data are means of four independent experiments carried out in triplicate. P = 0.0004. **C. Summary of quantification of cell-associated Env in Dlg1+ and Dlg1- cells and of virus-associated Env in progeny viruses.** The cell-associated Env levels were estimated from the intensity of the signals on western blots and calculated as gp160+gp120/Gag in cell lysates. Equal amounts of total protein in lysates of Dlg1+ and Dlg1- HIV-1-infected cells recovered 5 dpi were analyzed using antibodies against HIV-1 Gag and Env. The virus-associated Env levels (gp120) were estimated from the intensity of the signals on the western blots; equal amounts of p24 of purified viruses from supernatants of Dlg1+ and Dlg1- HIV-1-infected cells were analyzed using antibodies against HIV-1 Gag and Env. The infectivity of these viruses was determined as described in B. The data are from four independent experiments. **D. Western blot analysis of protein extracts of Dlg1+ and Dlg1- cells and of purified viruses produced.** Results of experiment 4 are shown. Left panel: equal amounts of total protein in lysates of Dlg1+ and Dlg1- HIV-1-infected cells recovered 5 dpi were analyzed using antibodies against HIV-1 Gag and Env, against Dlg1 and against GAPDH. Right panel: equal amounts of p24 of purified viruses from supernatants of Dlg1+ and Dlg1- HIV-1-infected cells were analyzed using antibodies against HIV-1 Gag and Env. **E. Cell-surface level of HIV-1 Env on Dlg1+ and Dlg1- HIV-1-infected T cells.** The cell-surface level of HIV-1 Env was determined by flow cytometry analysis using the anti-Env 5F7 antibodies. HIV-1-infected Dlg1+ and Dlg1- Jurkat T cells were analyzed 5 dpi. Two independent experiments were carried out in duplicate. Error bars represent SEM. ns  =  no statistically significant difference. *, P<0.05. **, P<0.01. ***, P<0.001.

The role of Dlg1 in cell-free virus transmission was further examined by studying the late steps of the viral life cycle such as virus yield and infectivity. HIV-1 yield during infection was followed in Dlg1+ and Dlg1- Jurkat T cells infected with HIV-1 by measuring by ELISA at different times the levels of p24 released in the supernatants. Virus yield was increased at 5 and 7 dpi in the Dlg1- Jurkat T cell culture compared to the Dlg1+ Jurkat T cell culture (Figure 4A).

The infectivity of viral particles produced by Dlg1- and Dlg1+ T cells was determined by their capacity to infect HeLa CD4+ indicator target cells. HIV-1 particles produced by Dlg1- cells 7 dpi showed a fourfold increase in infectivity compared to the particles produced by the Dlg1+ cells (Figure 4B). This study performed using stably Dlg1-depleted T cells infected with HIV-1 confirmed previous studies performed with adherent 293T cells depleted of Dlg1 by siRNA and transfected with a proviral HIV-1 plasmid [18]. Collectively, the results of virus yield and infectivity indicate that the enhanced HIV-1 replication observed in Dlg1- T cells (Figure 1C) results from a combination of increased yield and Dlg1 modulation of cell-free virus transmission.

To determine if the increased infectivity of viral particles produced by Dlg1- cells results from a higher incorporation of Env into viral particles, western blot analyses were performed on protein extracts of HIV-1-infected Dlg1+ and Dlg1- cells and of the viruses they produced. Figure 4C summarizes the results of four independent experiments. The amount of intracellular Env in Dlg1- cells compared to level in Dlg1+ cells was two to sixfold higher (gp160+gp120 normalized with respect to Gag). Whereas the amount of virus-associated Env in the viruses produced by Dlg1- T cells compared to that in the virus produced by Dlg1+ cells was halffold lower to threefold higher (gp120 normalized with respect to Gag). Infectivity of viruses produced by Dlg1- cells was increased from two to sixfold while the extinction of Dlg1 in these cells was from 50 to 80%. The results of experiment 4 (Figure 4C and D) show that Dlg1 depletion did not affect Gag production in Dlg1- T cells (Pr55Gag and p24; Figure 4D left panel) and that while a sixfold increase in intracellular Env was observed in Dlg1- T cells, the viruses produced by these cells showed 50% reduction of virus-associated Env and a fivefold increase in infectivity as compared to viruses produced by Dlg1+ T cells. In summary, Dlg1 depletion in T cells does not affect Gag synthesis but enhances cell-associated Env and virus infectivity. On the other hand, the changes observed on virus-associated Env did not always correlate with enhanced infectivity. To determine if the Env level at the plasma membrane is affected by Dlg1, the cell-surface level of HIV-1 Env was quantified by flow cytometry. No difference in Env cell-surface levels in HIV-1-infected Dlg1+ and Dlg1- Jurkat T cells was observed (Figure 4E). Consequently, viral particles budding at the plasma membrane of Dlg1+ and Dlg1- cells where Env levels are similar may incorporate similar levels of Env. Collectively, the results indicate that the increase in infectivity of particles produced by Dlg1- T cells does not correlate with an increase in levels of Env incorporated in these particles and that therefore virus-associated Env level alone is not responsible for the observed increase in infectivity.

### Subcellular distribution of HIV-1 particles in Dlg1-depleted T cells

**Figure 5:**
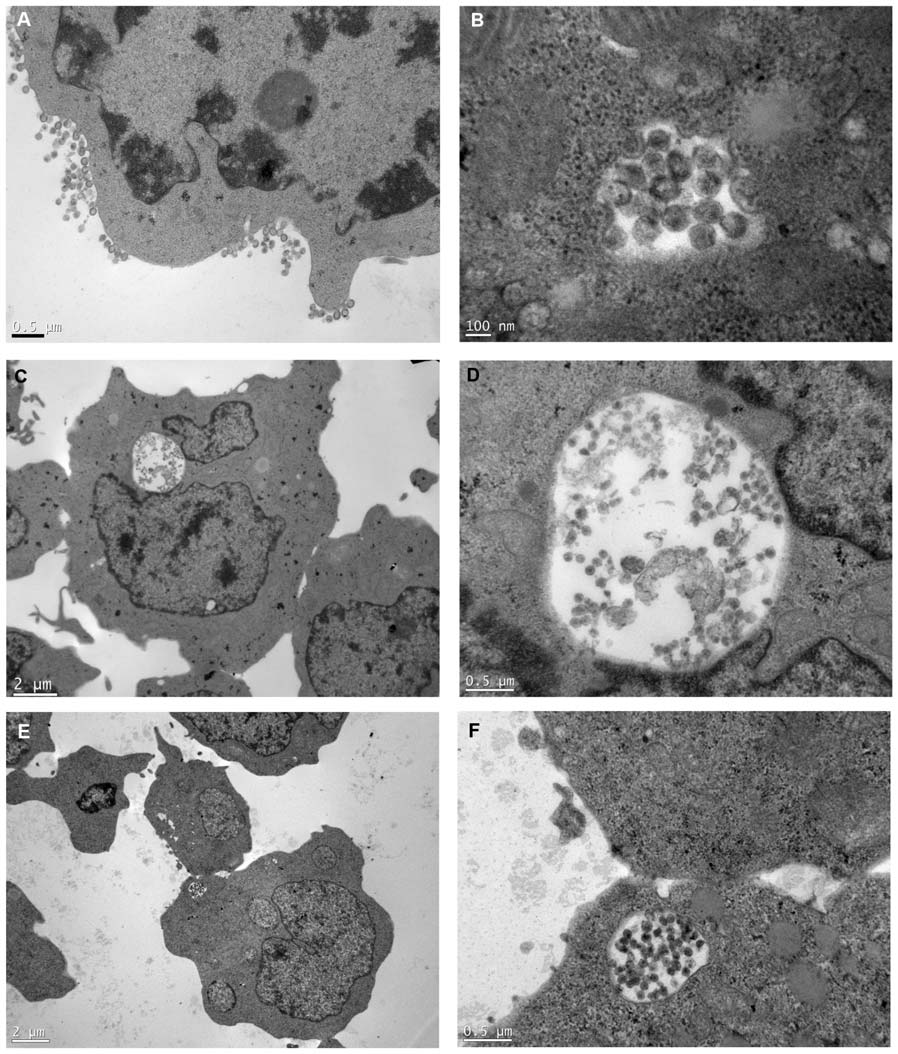
Budding of HIV-1 particles in Dlg1- Jurkat T cells occurs at the plasma membrane and in internal compartments. **A. Budding of HIV-1 particles in Dlg1- Jurkat T cells from the plasma membrane.** Dlg1+ and Dlg1- Jurkat T cells infected with HIV-1 were examined 6 dpi, when infection was about 60%, by electron microscopy in cells not forced to form conjugates. A total of 320 Dlg1+ Jurkat T cells (out of 5300) showing viral particles and of 392 Dlg1- Jurkat T cells (out of 6500) showing viral particles were counted. Budding from plasma membrane was identical in Dlg1+ and Dlg1- cells; the image presented is of a Dlg1- Jurkat T cell. Two independent experiments were performed with both NL4.3 and LAI.2 HIV-1. **B. Budding of HIV-1 particles in Dlg1- Jurkat T cells from internal compartments.** Cells were treated as above. Budding and mature particles are seen in an internal compartment in a Dlg1- Jurkat cell. **C and D. HIV-1 particles in internal compartments of Dlg1- Jurkat T cells.** Mature particles are seen in the enlargement in D. **E and F. HIV-1 particles in compartments near the plasma membrane of Dlg1- Jurkat T cells.** Mature particles are seen in the enlargement in F. Same magnification was used in C and E and in D and F. P = 0.02 for NL4.3. P = 0.01 for LAI.2 infected cells. Scale is 0.5 µm for A, D and F, 2 µm for C and E and 100 nm for B.

In a previous study we observed by confocal microscopy that Gag and Env were relocalized to apparently internal compartments in unconjugated Dlg1- T cells transfected with LAI.2 HIV-1-proviral clones [18]. To extend those studies with stably depleted Jurkat T cells infected with HIV-1, we examined by electron microscopy the subcellular distribution of viral particles in unconjugated cells. Cell cultures with a minimum of 60% infected cells were used for these experiments. Viral particles were seen in 6% of the cells by electron microscopy (i.e. in 10% of the infected cells). Viral particles budded exclusively from the plasma membrane in all virus-producing Dlg1+ Jurkat T cells as well as in 96% of Dlg1- Jurkat T cells (Figure 5A). Viral particles were seen in internal compartments in the remaining 4% of the virus-producing Dlg1- Jurkat T cells (Figure 5B to F). Interestingly, particles in the course of budding were observed in some of these compartments (Figure 5B). Two types of compartments were observed, large ones close to the nucleus (Figure 5C and D) and small ones near the plasma membrane (Figure 5E and F). The small ones may be invaginations of the plasma membrane, whereas the large ones are most likely internal compartments based on their size and localization inside the cell. Finally, 4% of virus producing cells of both Dlg1+ and Dlg1- cells showed small shallow invaginations (data not shown). No large deep invaginations were observed in any of the two types of cells. Similar results were obtained for Jurkat T cells infected with pNL4.3 and LAI.2 HIV-1. Statistical analyses using the chi2 test indicate that the presence of virus-containing internal compartments is significantly due to the absence of Dlg1 in the infected cells (P = 0.02 for pNL4.3 and P = 0.01 for LAI.2 infected cells). These electron microscopy studies show for the first time virus-containing internal compartments in acutely HIV-1-infected Dlg1- Jurkat T cells. They confirm our previous observations with confocal microscopy [18] and open the way for future characterization of the nature of the internal compartments as well as the elucidation of the role of these compartments in HIV-1 trafficking in T cells.

### Role of Dlg1 in cell-free virus transmission

**Figure 6:**
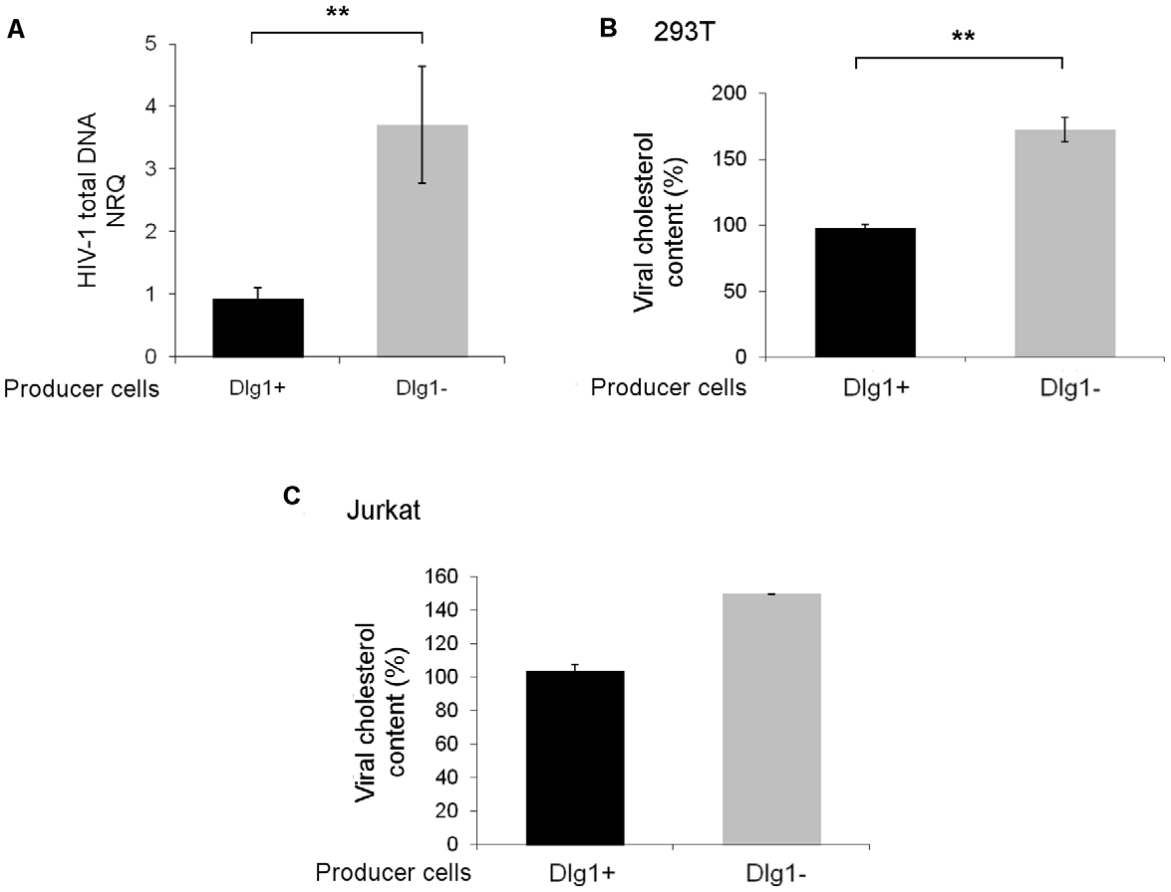
HIV-1 particles produced by Dlg1-depleted cells accumulated higher total HIV-1 DNA amounts during the first hours of infection and have increased cholesterol content. **A. Quantification of** t**otal viral DNA.** Total viral DNA was quantified by quantitative PCR in Jurkat T cells 6 h after infection with NL4.3 or LAI.2 HIV-1 particles produced by Dlg1+ and Dlg1- cells. The results are expressed as mean NRQ (normalized RQ described in materials and methods). Values are from one representative experiment performed in triplicate for infection and in triplicate for qPCR. P = 0.001. **B. Viral cholesterol content of HIV-1 particles produced by 293T cells.** Dlg1- 293T cells were obtained by siRNA transfection. The cholesterol content of LAI.2 HIV-1 produced by Dlg1+ or Dlg1- 293T cells was determined. The data are the means of three independent experiments performed in duplicate. For each experiment the values were normalized to the value obtained with one of the duplicates of viruses from Dlg1+ cells taken as 100%. P = 0.0026. **C. Viral cholesterol content of HIV-1 particles produced by Jurkat T cells.** Cholesterol content of LAI.2 HIV-1 produced by Dlg1+ or Dlg1- Jurkat T cells was determined. The data are the means of one experiment performed in duplicate. The values were normalized to the value obtained for one of the duplicates of viruses from Dlg1+ cells taken as 100%. Error bars represent SEM. **, P<0.01.

To investigate at which step Dlg1 affects infectivity of cell-free virus, total viral DNA was determined by quantitative PCR of HIV-1 DNA, 6 h after infection of Jurkat cells with NL4.3 HIV-1 produced by Dlg1+ or Dlg1- cells. A fourfold accumulation of total viral DNA was observed in Jurkat cells infected with virus produced by Dlg1- cells compared to Jurkat cells infected with virus produced by Dlg1+ cells (Figure 6A). Similar results were obtained when infections were performed with LAI.2 HIV-1. Thus the effect of Dlg1 on virus infectivity takes place at early steps of the virus life cycle, either entry or reverse transcription.

Cholesterol plays a key role in the HIV-1 life cycle; it was shown that viral infectivity correlates with viral cholesterol content and that virus-cell fusion capacity was directly linked with viral cholesterol [41]. Since Dlg1 coordinates the aggregation of lipids rafts during IS formation [24], the cholesterol content of viral particles produced by Dlg1 + and Dlg1- cells was examined. The average cholesterol content of LAI.2 HIV-1 particles produced by 293T cells depleted of Dlg1 was increased 1.7 fold compared to the cholesterol content of particles produced by control Dlg1+ cells (Figure 6B). This increase was in excellent correlation with the enhancement of infectivity of 1.8 fold observed for these particles. Similarly, the cholesterol content of the viruses produced by LAI.2 HIV-1 infected Dlg1- Jurkat T cells showing a 1.5 fold increased infectivity was increased 1.5 fold compared to the cholesterol content of the viruses produced by Dlg1+ cells (Figure 6C). These results show a robust correlation between the increase in cholesterol levels of HIV-1 particles produced by Dlg1- cells and their increased infectivity.

In conclusion, the increased accumulation of total viral DNA a few hours after infection with particles produced by Dlg1- cells, together with the increased cholesterol content of these particles and the fact that viral cholesterol content has been reported to determine fusion capacity of HIV-1 [41], are good indications that the effect of Dlg1 on HIV-1 infectivity is probably at the step of virus-cell fusion.

## Discussion

Dlg1 organizes signaling complexes at cellular contacts and particularly in T cells at the IS, where it is involved in the assembly and localization of TCR proximal signaling molecules and in the link between the TCR signaling machinery and regulators of the cytoskeleton [20], [23], [24]. The possible implication of Dlg1 in the VS had not been studied previously. We show here by IF and confocal microscopy studies that Dlg1 is involved in IS formation, but its depletion in T cells does not prevent conjugate formation or HIV-1-induced formation of both monosynapses and polysynapses. In addition, co-culture studies show that Dlg1 does not affect HIV-1 cell-to-cell transfer or effective virus transmission in T cells. Taken together, our results show that despite its key role in IS formation and T cell signaling, Dlg1 is dispensable for HIV-1 induced VS formation in T cells. The different role of Dlg1 in the IS and the VS, is consistent with the recent conclusion that while HIV-1 uses common structural components of the IS to form the VS, the latter has different morphological features, dynamics, intracellular signaling events and cytoskeleton reorganization [42].

Dlg1 is the only protein of the MAGUK family that has been identified and extensively studied in T cells [34] and the shRNAs used here specifically target Dlg1 but not the other proteins of the family. Therefore, it cannot be excluded that other proteins of this family could be present in T cells and compensate for the lack of Dlg1. We provide evidence that in stably depleted Dlg1 T cells the infectivity of the viral particles produced is enhanced. Since specific knockdown of Dlg1 is sufficient to affect HIV-1 particle infectivity, this indicates that in T cells Dlg1 is the main protein of the Dlg family involved in modulation of HIV-1 infectivity.

Our results show that HIV-1 infection of Dlg1- Jurkat T cells results in progeny viruses with enhanced infectivity and that in these cells the early steps of the HIV-1 life cycle are not affected. The absence of correlation between the level of virus-associated Env and the increase in virus infectivity observed in the present study is in contrast with our previous report [18]. The reason for this discrepancy is unknown; it may be attributable to differences between stable and transient depletion, transfection and infection or to cell type-specific differences. In our previous studies, biochemical analyses were performed in readily transfected 293T cells, rather than in T cells that are targets for productive HIV-1 infection as used in the present study. The absence of correlation between the level of virus-associated Env and the increase in virus infectivity is in agreement with a report where HIV-1 infectivity was not modified by a threefold increase in virus-associated Env content [43]. In addition, it was observed for *Simian immunodeficiency virus* (SIV) that a fiftyfold increase in virus-associated Env content only increased infectivity to two to threefold [44]. It was proposed that the effects of virus-associated Env content on infectivity are likely to depend on the SIV strain, the cell line and the co-receptor used [44].

Here we present viral-containing internal compartments in acutely HIV-1-infected Dlg1- Jurkat T cells. These exciting results open the way for the determination of the nature of these internal compartments as well as for the elucidation of their role in HIV-1 trafficking in T cells. Are these compartments late endosomes/multivesicular bodies as described in macrophages [45], [46], [47] and in chronically infected T cells [48] or are they invaginations of the plasma membrane as proposed for macrophages [49], [50]? What is the kinetics of formation of these compartments? Viral particles budding into these compartments were observed, indicating that at least some of these compartments are sites of particle assembly. Are these sites where more efficient assembly of virus particles occurs? It will be interesting to compare the infectivity, protein composition and cholesterol content of viral particles from the internal compartments to those from the supernatant of cell cultures at different times during infection, in order to elucidate the role of the internal compartments in the assembly of infectious HIV-1 virions.

The lipids of enveloped viruses play a key role in viral morphogenesis and infectivity and many viruses modify host cholesterol metabolism to optimize their replication. Increased infectivity of viral particles produced by Dlg1- T cells may result from different compositions of lipids of the host membrane microdomains from which virus budding occurs. Dlg1 coordinates synaptic lipid raft clustering during IS formation [24] and as a scaffold protein, it may alter the composition of lipids or proteins of the membranes where HIV-1 buds, acting on their incorporation into viral particles. HIV-1 buds from cholesterol rich membrane microdomains [51]. The overall lipid composition of HIV-1 resembles detergent-resistant membrane microdomains [52]. In addition, HIV-1 Nef enhanced the cholesterol content of HIV-1 progeny virions and increased their infectivity, while inhibiting the activity of the major cellular cholesterol transporter, the ATP binding cassette transporter A1 (ABCA1) [53]. Interestingly, it was reported that Dlg1 interacts with ABCA1 [54]. Reduced HIV-1 fusion activity was directly linked to reduced viral cholesterol content [41]. The Membrane Proximal External Region (MPER) preceding the transmembrane domain of the HIV-1 gp41 subunit was shown to be required for fusion activity by destabilizing the viral envelope and MPER fusogenic activity is enhanced by cholesterol [55], [56]. In this context, our results of increased infectivity and increased cholesterol content of HIV-1 particles produced by Dlg1- cells, together with increased accumulation of total viral DNA a few hours after infection of T cells with particles produced by Dlg1- cells, indicate that the effect of Dlg1 on HIV-1 infectivity is probably at the step of virus-cell fusion. To test this hypothesis, numerous experiments were performed with pseudotyped viruses as well as using the fluorescence resonance energy transfer-based HIV-1 virion fusion assay [57]. In all experiments using these systems to produce viruses in 293T cells depleted of Dlg1, the virions produced were less infectious than the control ones produced by Dlg1+ cells (data not shown). This phenotype is the opposite of the one obtained with wild type HIV-1 and underlines the important role of Dlg1 in the production of infectious particles.

Alternatively, increased infectivity may result from the activity of host factor(s) differentially incorporated into viral particles in the presence or absence of Dlg1. Dlg1 is not incorporated into viral particles [18] and it has not been reported in proteomic studies of HIV-1 [58], [59],[60]. The HIV-1 proteome analyses have identified a vast number of host proteins incorporated into virions (http://web.ncifcrf.gov/research/avp/). In this list are included the cell surface receptor cluster of differentiation 2 (CD2) and Lck, two cell proteins with which Dlg1 interacts. CD2 was found differentially incorporated in virus from sooty mangabeys but not from rhesus macaques infected by SIV [61]. CD2 will be tested in future studies for its possible differential incorporation into HIV-1 particles produced by Dlg1+ and Dlg1- T cells that could affect HIV-1 binding to target cells. Proteomic and lipidomic analyses of highly purified HIV-1 particles produced by Dlg1+ and Dlg1- T cells are needed to determine the nature of the factor(s) increasing infectivity of particles produced in the absence of Dlg1.

Increased infectivity of the virus produced by Dlg1- cells does not result in increased cell-to-cell HIV-1 transmission in these cells, indicating that cell-to-cell HIV-1 transmission in co-culture can overcome barriers that efficiently limit cell-free transmission. Compared to viruses produced by Dlg1- cells, viruses produced by cells expressing endogenous Dlg1 may have either reduced stability, reduced affinity for target cells or may be less efficient in fusion and replication. We proposed that by controlling the cholesterol content of HIV-1 particles, Dlg1 may regulate the fusion capacity of the particles, and that when these viruses are shed at high concentrations into the cleft of the VS directly next to the target cell, multiple viruses may enter the target cell, overcoming the reduced fusion capacity of individual particles. That multiple copies of HIV-1 can be co-transmitted across a single VS and infect a target cell was recently demonstrated [62].

In conclusion, we demonstrate for the first time that despite the key role of the scaffolding protein Dlg1 in the IS formation and T cell signaling, Dlg1 does not affect the VS formation or cell-to-cell spread of HIV-1. We further demonstrate that Dlg1 plays a role in HIV-1 cell-free virus transmission and postulate that by modifying viral cholesterol content Dlg1 affects the step of virus-cell fusion. Our studies will have important implications for understanding how Jurkat and CD4+ primary T cells can rescue the deficiencies of cell-free virus by cell-to-cell transmission. Further insights into the role of Dlg1 on lipids or other host factors incorporation into HIV-1 may provide new ideas on the relationship between structure and function of infectious viral particles.
